# Pelvic Extirpative Surgery for the “End-Stage Irradiated Bladder”

**DOI:** 10.3390/cancers15174238

**Published:** 2023-08-24

**Authors:** Nikolas Moring, Seamus Barrett, Andrew C. Peterson, Brian M. Inouye

**Affiliations:** 1 Department of Urology, Albany Medical Center, Albany, NY 12208, USA; moringn@amc.edu (N.M.); barrets8@amc.edu (S.B.); 2 Department of Urology, Duke University, Durham, NC 27710, USA; drew.peterson@duke.edu

**Keywords:** cystectomy, extirpation, prostate cancer, radiation, fistula, osteomyelitis, cystitis, ileal conduit, reconstruction

## Abstract

**Simple Summary:**

Post-radiation prostate cancer patients are at an increased risk for developing a multitude of long-term complications, notably, a nonfunctional bladder with associated fistulae, bleeding, and infection. In its advanced stages, this can be difficult for the clinician to effectively manage. Our review discusses the role of cystectomy in patients with a radiation-induced end-stage bladder and the challenges that an irradiated surgical field poses to the surgeon. In summary, the literature supports cystectomy in select patients as an option for definitive treatment, which can drastically improve the quality of life in these patients.

**Abstract:**

Men with prostate cancer have the daunting task of selecting from multiple modalities of treatment. The long-term effects of radiation therapy are only now being recognized. For both patients and surgeons, the end-stage irradiated bladder poses numerous problems and challenges. Specifically, irradiated bladders with urosymphyseal fistula, radiation cystitis, and rectourethral fistula are challenging to manage and treat. This review outlines the presentation, workup, and management including cystectomy for these three devastating late complications of radiation therapy. There are special considerations when performing benign cystectomy that are not typically considered during oncologic cystectomy. We discuss an overview of the current literature regarding the “end-stage bladder” resulting from radiation therapy and the important considerations that must be acknowledged when managing these patients. It is shown that many of the less invasive and conservative options ultimately lead to cystectomy. Indeed, our review concludes that cystectomy with urinary diversion is a safe and viable option in select irradiated patients with the goal to improve quality of life.

## 1. Introduction

Pelvic extirpative therapy includes pelvic organ removal and urinary diversion and has been used by surgeons since its first description in 1887 by Dr. Bernhard Bardenheuer in Cologne [1]. Since then, its use has been extrapolated to treat urologic, gynecologic, and colorectal malignancies. Its use has also been described for benign conditions often seen in cancer survivors, such as the “end-stage bladder”, as a result of urosymphyseal fistula, radiation cystitis, and rectourethral fistula. The end-stage bladder can also occur in non-cancer survivors as a result of bladder outlet obstruction, interstitial cystitis, neurogenic dysfunction, and idiopathic causes [2].

Prostate cancer is the most common non-cutaneous malignancy in men and accounted for 27% of all new cancer diagnoses in men in 2022 [3]. Men diagnosed with prostate cancer are faced with the daunting task of selecting treatment for their malignancy from a variety of different modalities including surgery, radiation, prostate tissue ablation, and active surveillance when appropriate [4]. The multiplicity and variation in treatment approaches are leading to new post-treatment side effects, which we are only now beginning to understand. Additionally, the post-treatment side effects now being seen are likely subject to survivor bias: men are surviving much longer after excellent prostate cancer treatment, leading to side effects decades later [5]. These side effects were not well-described in initial shorter-term follow-up studies.

“End-stage bladder” is a term that encompasses a bladder with little to no meaningful function and/or the potential for harm. This includes bladders with significant fibrosis, fistula, small capacity, reduced compliance, poor contractility, incontinence, and often inflammation and pain [6]. For these patients, bladder dysfunction does not encapsulate the full gravitas of their disease, and a more appropriate term would be “bladder failure”. One of the most serious complications of an end-stage bladder includes upper tract deterioration [7]. The term “voiding dysfunction” is also not appropriate for these patients as this fails to capture the far-reaching and multifaceted symptomatology of end-stage bladder disease [8].

Organ preservation using augmentation cystoplasty is one management option for an end-stage bladder. Though this often achieves the goal of producing a large-capacity, low-pressure urinary reservoir, up to 48% of patients are still dissatisfied after augmentation cystoplasty for end-stage bladder [2]. Even after augmentation cystoplasty in a well-selected patient population, up to 5% will need further diversion surgery afterward due to failure of the reconstructed anti-reflux mechanism [9].

Furthermore, a radiation-induced end-stage bladder may present with concurrent urosymphyseal fistula, radiation cystitis, or rectourethral fistula and may also warrant pelvic extirpative therapy. It is currently estimated that between 5.4 and 8% of cystectomies performed in the United States are for benign indications [10]. Though cystectomy and diversion has been well studied in the oncologic spheres, their application in benign disease is still being investigated [11]. We aim to provide a succinct but comprehensive overview of pelvic extirpative therapy including cystectomy and incontinent urinary diversion for patients suffering from an end-stage irradiated bladder and its non-oncologic complications.

## 2. Pubic Osteomyelitis with Urosymphyseal Fistulae

Pubic osteomyelitis with urosymphyseal fistula in prostate cancer survivors is becoming a well-defined clinical complex that typically develops a decade or more after prostate cancer treatment [12]. Presentation is variable but commonly consists of symptoms such as pelvic pain, difficulty ambulating, and urinary incontinence. Typically, these men have a history of external beam radiotherapy and endoscopic outlet procedures to treat outlet obstruction from scar tissue after radiation therapy as part of their prior prostate cancer treatment. While initial attempts at conservative management (antibiotics, drainage) may temporize these patients, antibiotics typically have very poor penetrance of the bone, necessitating cystoprostatectomy, diversion, and pelvic bone debridement for definitive treatment [13].

**Figure 1:**
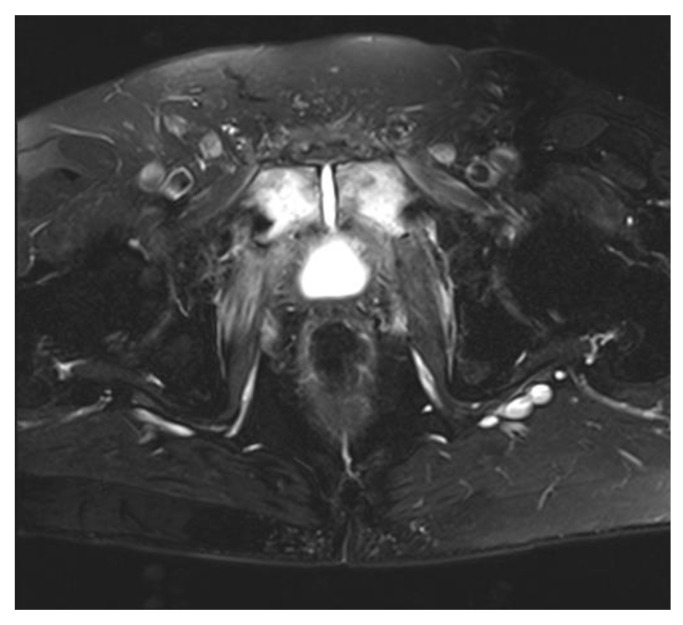
A pelvic MRI of a patient with pubic osteomyelitis. This T2-weighted image is an axial view of the pelvis, which shows an increased signal (bright white) in the bladder as well as the pubic symphysis and surrounding bone consistent with osteomyelitis from a urosymphyseal fistula. Courtesy of Brian Inouye.

The initial workup should include a thorough history and physical, with special attention paid to cancer diagnoses and treatments, including time since diagnosis and the use of radiation, if applicable. Patients may have had prior endoscopic treatment such as direct vision internal urethrotomy (DVIU) or bladder neck incision (BNI). Patients should get a standard set of labs and PSA to check for recurrence of prostate cancer as well as inflammatory markers such as the erythrocyte sedimentation rate (ESR) and C-reactive protein (CRP) along with the white blood cell (WBC) count. Nutrition studies such as pre-albumin may also be helpful in assessing overall nutritional status, as many patients are cachectic from a chronic inflammatory state [14]. A urine culture is recommended, and if symptomatic, treatment is warranted. The majority of patients will need a pelvic MRI to look for the presence of chronic osteomyelitis at the symphysis pubis and to image any possible urosymphyseal fistula (Figure 1). All patients should also undergo cystoscopy to evaluate the bladder and urethra for other pathology. Other pre-surgical interventions can include hyperbaric oxygen, nutritional counseling and supplementation, and cessation of smoking.

In a cancer survivor, this condition can be an intractable source of pelvic pain and debilitation and cause a loss in quality of life [15,16]. As previously mentioned, conservative regimens fail to cure bone infection in the majority of patients, and surgical correction is required [17]. These patients often had concomitant treatment for bladder neck contractures prior to their diagnosis of urosymphyseal fistula, making reconstruction of the posterior urethra difficult [18]. While there have been attempts at augmentation and preservation of the orthotopic bladder with volitional voiding, contemporary series have focused on pelvic extirpation consisting of pubectomy along with cystectomy and urinary diversion. This approach generally results in an improvement in patients’ overall quality of life and pain, and yields increased physical functioning and a return to activities of daily living [8,16]. A recent retrospective study suggested that pelvic extirpation reduced long-term pain and long-term opioid use in this patient population [19].

**Figure 2:**
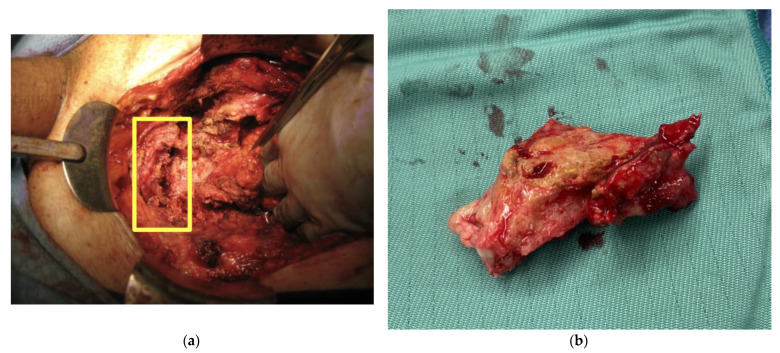
(**a**). Pelvic bone debridement during pubectomy for pubic osteomyelitis. Intraoperative photograph showing pubic symphysis after bone resection. The yellow box shows area of pelvic bone removal. Courtesy of Brian Inouye. (**b**) Resected bone. Intraopeartive specimen of bone removed from a patient that was found to be affected by chronic osteomyelitis. Courtesy of Brian Inouye.

The operative field is typically plagued by the sequelae of radiation including devastation of tissue planes, making dissection quite difficult. The goal of surgery is to remove the bladder and fistulous tract along with the affected bone and create a urinary diversion—oftentimes, an ileal conduit (Figure 2). Although continent diversions such as a catheterizable pouch may be appropriate, orthotopic continent diversions are not indicated in these situations due to the radiation changes in the pelvic floor. Colorectal surgery consultation may be necessary if planes near the rectum are difficult to distinguish if there is any suspicion of bowel involvement with a rectourethral fistula. Furthermore, most modern series discuss supratrigonal cystectomy as an alternative to a radical cystectomy in order to minimize intraoperative rectal injury. Orthopedic surgery may need to be consulted for assistance with pubectomy, depending on the surgeon’s experience. Bone cultures should be sent at the time of surgery after holding antibiotics for two weeks preoperatively to provide for a non-sterile specimen [13].

It is the authors’ opinion that proper handling of the inferior rectus abdominus and its fascia is of critical importance during this surgery. As the pubic symphysis and lateral superior rami are typically excised, there is a loss of inferior attachments to the rectus abdominus. Splitting of the fascia and underlying muscle with the removal of the underlying pubic symphysis may lead to a difficult-to-treat penile hernia. We believe one solution is to leave the inferior rectus fascia attached in the midline and roll the fascia and underlying muscle off the pubic symphysis to remove the bone while keeping the overlying musculature intact. Other described techniques to prevent penile hernia following pubectomy include the vertical rectus abominis musculocutaneous (VRAM) flap [5]. In cases where a VRAM flap is not used, omentum is generally the preferred tissue for flap coverage. Even if the inferior rectus fascia is kept intact, we agree that some sort of flap (typically posterior peritoneum or omentum) should be used to fill in the pelvic dead space to prevent empty pelvis syndrome and bowel adhesion to the exposed bone.

Pre-operatively, we often have patients meet with our wound ostomy care nurses for consultation and stoma marking. During their inpatient stay, there is ample time for them to receive stoma teaching, and we feel it is important that patients and family members participate in learning stoma care prior to discharge.

Post-operatively, nutritional optimization is paramount as many times these patients have poor pre-operative nutritional status due to their chronically infected, catabolic state. Early mobilization and work with physical and occupational therapy are also of utmost importance as patients are at risk of deconditioning [20,21]. We emphasize a non-narcotic pain control regimen using ERAS (enhanced recovery after surgery), which may lead to shorter hospital stays and less post-operative ileus [22].

## 3. Radiation Cystitis

Another population of irradiated patients is those with refractory hemorrhagic/radiation cystitis. Unlike pelvic extirpative therapy for pubic bone osteomyelitis, this population of patients is mainly present due to a loss in quality of life, usually in the form of recurrent admissions to the hospital for refractory gross hematuria and anemia. Due to the recurrent nature of the disease course, radiation cystitis can be very burdensome to the patient, resulting in an increased likelihood of transfusions and operative intervention as well as increased healthcare costs [23].

Radiation cystitis has a reported incidence of 23–80% in patients who have undergone pelvic radiation therapy for all forms of pelvic malignancy, with the incidence of severe hematuria ranging from 5 to 10% [24]. The mean time to develop radiation cystitis is 31.8 months following treatment and can develop many years later [24]. Turchan et al., 2022 found that hematuria occurred in 35% of their patient cohort who had undergone post-prostatectomy radiation therapy, with 7% ultimately requiring intervention [25]. Another study found the incidence of hemorrhagic radiation cystitis to be 11.1% in prostate cancer patients who underwent radiation therapy, with 52% requiring blood transfusions [26].

It is suggested that damage to the microvasculature and urothelium during radiation may lead to a cascade of fibrosis, oxidative stress, and inflammation. This process ultimately results in increased edema, ulceration, and aberrancy in the cellular microenvironment. In the acute phase, symptoms may include increased urinary frequency, dysuria, and a low-capacity bladder. There may also be a latent phase that may last from months to years, characterized by endarteritis and a leaky urothelium, at which point the patient may be asymptomatic. The late phases of the disease are characterized by hemorrhage and edema, at which point troublesome hematuria becomes a prominent symptom [27,28].

The Canadian Urological Association published a best practice report in 2019 outlining their recommended workup and management for radiation cystitis. It is necessary for a clinician to obtain a detailed oncologic history, particularly regarding previous radiation treatment dosage and course, in addition to a complete history and physical. Initial management of bleeding typically requires large bore foley catheter placement, manual irrigation for clot removal, hydration with fluids, and often continuous bladder irrigation (CBI). Consideration can be given to taking the patient to the operating room for cystoscopy and clot evacuation with fulguration of bleeding [29].

In cases where bleeding is refractory to irrigation, a variety of intravesical agents may be considered. Alum irrigation can be considered by acting as an astringent on the cell surface, resulting in decreased capillary permeability, vasoconstriction, and hardening of the intercellular space [30]. Side effects include bladder spasms, precipitation of sediment that may clot the catheter, and aluminum toxicity. Hyaluronic acid is a mucopolysaccharide that can also be considered as an intravesical agent, although it may be more effective in treating LUTS rather than hematuria in radiation cystitis [31]. Aminocaproic acid (Amicar) was shown to improve hematuria in a case series in 1992 when used as an intravesical agent in hemorrhagic cystitis, but the literature has been limited regarding its utility since then [32]. Formalin is sometimes utilized as a last-resort intravesical therapy due to its potential complications. The administration can be painful for a patient, so formalin instillation should be performed under anesthesia. Its instillation results in capillary occlusion at the level of the urothelium, preventing further bleeding. Vesicoureteral reflux should be excluded prior to administration to prevent damage to the ureters or kidneys [33]. Vesicoureteral reflux can be assessed intraoperatively by performing a cystogram prior to the administration of formalin. However, vesicoureteral reflux is not an absolute contraindication to formalin administration; if identified, ureteral occlusion balloons can be placed, and formalin administration can proceed.

Hyperbaric oxygen treatment (HBOT) as a long-term treatment has also been demonstrated to improve symptoms of late radiation cystitis compared to controls [34]. Factors including an increased number of HBOT sessions, younger age, lower radiation dose, and faster initiation of treatment after the start of symptoms have been associated with increased success [35]. Hyperbaric oxygen treatment is a good option for patients who have access to it; however, HBOT centers may not be easy for all patients to access and require frequent appointments to maximize benefits, making the care sometimes burdensome for patients [36]. There may also be significant associated costs with HBOT, rendering its use prohibitive at times, though Medicare Part B does cover HBOT for radiation injuries [37]. Cystectomy and urinary diversion may be considered as the last line of management if bleeding continues to be refractory.

Pre-operative workup should include a thorough history and physical. Special attention should be paid to the number of admissions for gross hematuria and prior need for blood transfusions, the use of intravesical agents, and other palliation methods. Patients should get a pre-operative complete blood count and basic metabolic panel, as well as anemia studies. It is imperative that patients have a urine culture prior to surgery and should receive treatment if infection is present. Preoperative cystoscopy should also be performed.

It is important to note that when leaving the bladder in situ with supravesical diversion, severe complications may still occur including bladder pain, bleeding, and pyocystis [38]. Additionally, it is estimated that men treated with radiotherapy have a 3% chance of secondary primary malignancy, and this risk increases over time [39]. Secondary bladder cancer may present concomitantly with radiation cystitis and with more aggressive features [40,41]. One study found that in 30 patients who underwent supravesical diversion for benign disease, 80% experienced at least one complication related to the in situ bladder, with 43% requiring rehospitalization [42]. Due to these potential complications, a refractory patient will need a cystectomy with incontinent urinary diversion.

This patient cohort is of high operative risk, instigated by an irradiated surgical field. Cystectomy in this case should be carried out at a center of excellence with experience in complex reconstructive diversion. The history of radiation in these patients makes dissection difficult, and tissue planes may be completely devastated. It is the authors’ recommendation that pre-operative discussion with colorectal surgery should occur in case the need for intraoperative assistance arise due to difficulty with the posterior dissection.

Linder et al. 2014 evaluated 21 patients who underwent cystectomy with urinary diversion for hemorrhagic cystitis. In their study, they found Clavien grade III-V complications in 42% of the patients, with a 90-day mortality rate of 16% [43]. Although the operative risk may be significant, it has been suggested that cystectomy can significantly improve these patients’ health-related quality of life postoperatively. Most notably, there was improvement shown in reported levels of pain control and mental well-being [44]. It has also been suggested in a more recent retrospective study that at one high-volume center, cystectomy with diversion for benign disease in patients with a history of radiation did not increase perioperative morbidity when compared to those who underwent diversion without cystectomy [45].

## 4. Rectourethral Fistula

Rectourethral fistula (RUF) is an uncommon and devastating complication that can occur following prostate cancer treatment [46]. In the context of pelvic extirpative surgery, RUF is often seen as a complication of prostate cancer and anorectal cancer treatment. In the case of prostate cancer patients, it can present either shortly after surgical resection of the prostate, especially if the injury is unrecognized or as a delayed diagnosis after radiation treatment. It has been well demonstrated that the incidence of RUF is increasing and may correlate with the increased use of radiation in this population [47]. The incidence of RUF after prostatectomy appeared to decrease with the use of robotic radical prostatectomy, and it is currently estimated that <1% of all prostatectomies result in RUF. The addition of salvage radiation, salvage prostatectomy, prior rectal surgery, and transurethral resection appear to increase the risk of RUF [48]. Rectourethral fistula has even been documented with the use of SpaceOAR, an FDA-approved hydrogel designed to create space between the prostate and rectum during prostate radiotherapy. A study in 2019 that reviewed the Manufacturer and User Facility Device Experience (MAUDE) database found rectourethral fistula occurred in four cases, all of which required diverting colostomy [49].

Patients presenting with RUF often have pneumaturia, fecaluria, and a urinary tract infection. Workup and diagnosis of RUF have been described in an algorithmic approach by Hanna et al. [47]. This includes a thorough history and physical, with attention to cancer history and treatments, if applicable. It has also been recommended to obtain a baseline voiding diary, 24 h pad weight [50]. Standardized questionnaires have been recommended, which include the International Index of Erectile Function-5 score, the International Continence Society Male Short Form Questionnaire, and the American Urological Association Symptom Score. Physical examination should include a digital rectal exam with careful attention paid to the anterior rectal wall to palpate any fistula tracts. Imaging studies should include a voiding cystourethrogram or retrograde urethrogram, which may provide valuable information on fistula size and location. If patients are candidates for bladder-sparing surgery, urodynamic studies may be indicated to confirm a low-pressure, adequate-volume reservoir exists. Patients then often proceed to the operating room for cystoscopy as well as sigmoidoscopy and exam under anesthesia. This is also an opportunity to perform biopsies if there is a suspicion of secondary malignancy, as it is paramount to rule out the recurrence of malignancy [51]. It is important for the urologist to perform upper tract imaging (typically, a retrograde pyelogram) to rule out concomitant ureteral injury. Many surgeons choose to place a suprapubic tube during this time to divert the urine away from the fistula. Many patients may benefit from preoperative nutritional evaluation and counseling, as they are typically in a chronically infected state that may negatively impact their nutritional status.

While not the focus of this article, we think it is important to highlight that successful rectourethral fistula repair is often a multistep process involving four key operations. First, patients must have a urinary and fecal diversion to allow the fistula to mature in the diverted environment. Fecal diversion is often advised but may not be necessary in non-irradiated patients [52]. Secondly, patients have their fistula repaired. The third operation includes fecal and urinary un-diversion if applicable. However, most patients will need a fourth operation to address the resultant stress urinary incontinence [53]. During the counseling process, it is important to inform patients that while the goal is fistula repair and un-diversion, studies have shown that up to 10% of patients may need to be permanently diverted [54]. It has been shown that there is a lower success rate of RUF repair in irradiated or ablated patients [55,56,57]. This has led some institutions to recommend hyperbaric oxygen therapy to all patients with a history of radiation or ablation, though its benefit has not been directly evaluated in RUF. If the bladder is salvageable and the RUF is deemed reconstructible, surgical treatment is a multidisciplinary approach and usually involves urology, colorectal surgery, and plastic surgery [58]. With colonic diversion, loop colostomy may be preferred to loop ileostomy given superior nutritional and fluid status. Diversion decreases inflammation and may help prevent further infection while patients are awaiting definitive RUF treatment. In rare cases of non-irradiated small fistula, management with diversion has resulted in fistula closure, though this is an exception and not the norm [59,60,61].

There have been multiple described approaches to repair the rectourethral fistula. The York Mason procedure involves a transrectal, trans-sphincteric approach with patients positioned prone on the operating room table. It is an effective and low-morbidity procedure, with low post-operative complication rates [62]. The York Mason approach has excellent success for small, non-irradiated fistulas, but its success decreases with larger, irradiated cases [47]. With the use of a Kraske laterosacral approach, one can even spare the rectal sphincter [63]. Many modern authors prefer a perineal approach to rectourethral fistula repair as this approach is familiar to urologists. One major advantage to the perineal approach is local access to a variety of interposition flaps including gracilis, dartos, and peritoneum [64,65]. Since irradiated fistulas are becoming more common, the need for an interposition flap for adequate blood supply and the barrier between the rectum and urethra makes this approach appealing. If there is concomitant urethral stricture or a large urethral defect, surgeons may supplement their repair with buccal mucosal grafts [46]. Transabdominal repairs have previously been described but have largely fallen out of favor.

While there are high success rates for reconstructing RUF in select patients, cystectomy and conduit may also be necessary for those that are not deemed reconstructible. This patient cohort includes those with a history of radiation and a RUF > 3 cm, irreparable tissues, a distal RUF, or poor rectal tone [50]. It appears that the most predictive factor for the need for diversion and pelvic extirpation is a history of radiotherapy or ablative procedures, and fistula size is less important [66]. As previously discussed, radiation can lead to a devastated bladder with high pressure and low volume storage. Additionally, the tissue in post-radiotherapy or ablation patients is less amenable to primary repair, which is in contrast to RUF in patients caused by surgery without radiotherapy. Case series of patients with irradiated rectourethral fistula have highlighted the failure of fistula repair in these patients and the need for permanent diversion [67]. The potential for tissue hypoxia and microvascular changes make repair more difficult. Delayed effects of radiation therapy also lead to changes in bladder compliance and continence. Patient selection and counseling are crucial. Patients may elect for cystectomy instead of fistula repair if the post-repair urinary system will be of low capacity or poor compliance; these problems often co-exist together.

The surgical field is often plagued by the sequelae of radiation with devastated tissue planes. If there have been no prior attempts at repair, patients may need a colonic diversion at the time of cystectomy. Often, these patients are treated using a stepwise approach with permanent diversion and cystectomy occurring after the previously described temporizing measures, though some patients may proceed directly to permanent fecal diversion if the fistula is deemed non-repairable.

## 5. Lessons Learned

The end-stage irradiated bladder is a difficult clinical scenario that requires careful consideration of treatment options, and there is truly no single best option for most patients. However, it is the authors’ opinion that several lessons have been learned as this field of reconstruction has grown.

1. First and foremost, not all bladders are salvageable for reconstruction, and patients should be counseled on this early in their workup. Radiation can have devastating side effects, rendering some bladders unsalvageable even in the most experienced of hands. Shared decision-making and informed consent are crucial.
2. During cystectomy, it is important to evaluate the ureters for changes secondary to radiation. A post-diversion ureteral stricture is a devastating complication that can render patients reliant on proximal diversion such as percutaneous nephrostomy if a less-than-optimal distal segment of the ureter is anastomosed to the conduit. We routinely inspect the ureters with intraoperative ureteroscopy to ensure the distal segment appears healthy and viable. Other groups have reported SPY or indocyanine green if being performed robotically [68]. If there is any suspicion of compromise, we excise the distal segment until healthy tissue is encountered. On the topic of ureters, we believe the Wallace ureteral anastomosis is superior to a Bricker anastomosis due to the lower rate of ureteroenteric stricture [69].
3. The ileum is nearly always the preferred bowel segment for conduit due to its ease of use, location, and short length, which makes it optimal for patients with renal impairment as it has the shortest contact time with urine [70]. A history of radiation should not preclude its use.
4. Lastly, these operations are quality-of-life operations, and this should be paramount in how one counsels their patient. A surgeon should always consider the surgical risk with the post-operative benefit. Intraoperative complications such as rectal injury may further worsen patient quality of life despite a surgical goal of improvement.

## 6. Conclusions

The irradiated bladder presents unique challenges for both the patient and surgeon alike. For patients, this often means months or years of debility, pain, frequent infections, and ultimately, prolonged hospitalization with one or more surgeries and a long road to recovery. Since there is no definitive best treatment for the irradiated, end-stage bladder, patients may find additional difficulty in the shared decision-making process when offered multiple approaches. For surgeons, the irradiated bladder represents a surgical conundrum of difficult dissection in a devastated field with a frail patient at risk for post-operative complications. Luckily, for many patients, surgery for the irradiated bladder, including extirpation, often leads to improvements in quality of life that could not be achieved otherwise. Of course, this is not true for all patients, and patients and surgeons should have a careful and considerate discussion about the risks, benefits, and alternatives to pelvic extirpative therapy. Patients and surgeons should carefully consider the multidisciplinary team involved in care, and many of these cases will need to be referred to a tertiary care center. However, in the correct patient, pelvic extirpative therapy for the end-stage, irradiated bladder can be a life-restoring treatment.

## Data Availability

No new data were created or analyzed in this study. Data sharing is not applicable to this article.
